# Accounting for temporal and individual variation in the estimation of Von Bertalanffy growth curves

**DOI:** 10.1002/ece3.9619

**Published:** 2022-12-21

**Authors:** Jasper Cornelis Croll, Tobias van Kooten

**Affiliations:** ^1^ Institute for Biodiversity and Ecosystem Dynamics University of Amsterdam Amsterdam The Netherlands; ^2^ Wageningen Marine Research Wageningen University and Research Wageningen The Netherlands

**Keywords:** environmental limitation, individual variation, length‐at‐age data, life history, North Sea plaice, population structure, Von Bertalanffy growth curve

## Abstract

Growth and growth limitation are important indicators of density dependence and environmental limitation of populations. Estimating individual growth trajectories is therefore an important aspect of understanding and predicting the life history and dynamics of a population. Variation in individual growth trajectories arises due to variation in the environmental factors limiting individual growth. This environmental limitation can vary over time, between cohorts and between individuals within a cohort. For a complete and accurate understanding of individual growth in a population, it is important to include all these sources of variation. So far, statistical models only accounted for a subset of these factors or required an extensive growth history of individuals. Here, we present a novel model describing the growth curves of cohorts in a population. This model is derived from a stochastic form of the Von Bertalanffy growth equation describing individual growth. The model is specifically tailored for use on length‐at‐age data in which the growth trajectory of an individual is unknown and every individual is only measured once. The presented method can also be used if growth limitation differs strongly between age or length classes. We demonstrate the use of the model for length‐at‐age data of North Sea plaice (*Pleuronectes platessa*) from the last 30 years. Fitting this model to length‐at‐age data can provide new insights in the dynamics of the environmental factors limiting individual growth and provides a useful tool for ecological research and management.

## Graphical abstract

Individual growth is often strongly affected by the environment. This could result in large variation in body size over time and between individuals and cohorts. We present a method to fit growth curves and estimate the environmental limitation of growth, while accounting for these sources of variation.

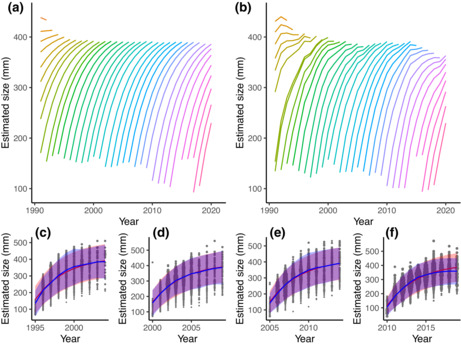

## 1 INTRODUCTION

Body length is shown to be an important indicator of many life history traits such as maturation, consumption, mortality, and reproduction rates (Beverton & Holt, 1959; Calder, 1984; Kooijman, 2010; Pauly, 1980; Peters, 1983). Estimating the growth trajectory of individuals is therefore a general aspect of understanding the life history and dynamics of a population. The Von Bertalanffy growth equation is one of the most commonly used models to describe growth of individuals. It is already used to describe the growth of a wide range of species (Kingsley, 1979; Narinc et al., 2017; Ramirez et al., 2021; Teleken et al., 2017) and is especially often used for fish (Flinn & Midway, 2021; Lorenzen & Enberg, 2002). The Von Bertalanffy growth equation describes growth in terms of individual energy assimilation and catabolism rates (von Bertalanffy, 1938). In the resulting growth curve, individuals grow toward an asymptotic length at which the catabolism rate is equal to the assimilation rate and no energy is available for growth. If we follow Dynamic Energy Budget theory (Kooijman, 2010) and assume that assimilation scales with surface area while catabolism scales with body volume, we obtain the most commonly used form of the Von Bertalanffy growth equation:

(1) $$\frac{d\ell_{\left(t,a,i\right)}}{\mathrm{dt}}=r_{B}\left(f_{t}\ell_{\infty }-\ell_{\left(t,a,i\right)}\right)$$

Herein, we indicate the length of individual *i* at age *a* and time *t* with $\ell_{\left(t,a,i\right)}$, which emphasizes that Equation (1) describes the growth rate of a single individual. In this equation, individual growth is proportional to the difference between the asymptotic length ($f_{t}\ell_{\infty }$) and the current length, scaled with the Von Bertalanffy growth rate scalar (*r*_*B*_). Following Dynamic Energy Budget theory (Kooijman, 2010), the asymptotic length consists of a maximum asymptotic length ($\ell_{\infty }$) scaled by the limitation of the environment through assimilation (*f*_*t*_). Although individuals might vary slightly in the values of the Von Bertalanffy growth rate scalar and the asymptotic size due to genetic differences, we will only focus on environmental effects on growth because these effects can affect the growth of individuals through their life. It is commonly assumed that the asymptotic length is the only parameter in the Von Bertalanffy growth equation which depends on the environment (Kooijman, 2010; Lorenzen & Enberg, 2002). As such, the Von Bertalanffy growth equation provides an opportunity to estimate the environmental limitation on the individual growth rate.

Research on the dynamics of individual growth is generally based on one of two types of data containing age and length measurements of individuals. The first type of data contains multiple measurements of the same individual, for example, obtained through controlled experiments, mark‐recapture methods or back calculation from otoliths or year‐rings. This type of data generally allows for extensive correction for variation between individuals and cohorts because the growth history of individuals is known (Graaf & Prein, 2005; Rafail, 1973; Shelton et al., 2013; Vincenzi et al., 2014, 2016). Often, however, such rich individual‐level data is unavailable, because individuals cannot be tagged or retrieved or because back calculation of otoliths and year‐rings is often imprecise (Eveson et al., 2007). Even if these methods are successful, they only result in a relative relationship between age and length. Much more common is the second type of data, which only contains a single measurement per individual. To obtain this kind of data, individuals only have to be measured once and the age of individuals can be determined based on hard or internal body structures such as year‐rings, scales, bones, teeth, and chemical composition. This is a common method for fish (Maceina et al., 2007), amphibians (Smirina, 1994), reptiles (Castanet, 1994), mammals (Read et al., 2018), and insects (Robson & Crozier, 2009). In this study, we focus on the estimation of growth curves and variation herein based on data consisting of a single observation per individual.

In data with a single age and length observation per individual, the growth history of individuals is unknown and it is difficult to deal with the different overlapping sources of variation in the length individuals have at a specified age. In addition to variation between measurements due to sampling errors (Piner et al., 2016; Taylor et al., 2005), variation in the growth rate can be separated into variation as a result of changes in the environment over time, variation in the growth history of cohorts, and variation between individuals within a cohort. So far, statistical methods dealing with single observations of individual ages and lengths only deal with a subset of these sources of variation.

Variation due to changes in the environment over time is most likely to affect the asymptotic length in the Von Bertalanffy growth equation because this is the part of the growth equation that is related to the environment‐dependent assimilation rate of an individual (Kooijman, 2010; von Bertalanffy, 1938). Changes in the asymptotic size therefore affect all cohorts at a given time equally. This can be used to estimate the effect of an environmental factor on the growth of individuals. This is generally done by directly substituting the asymptotic length in the Von Bertalanffy growth equation with a linear dependency on the environmental factor of interest (Cloern & Nichols, 1978; Graaf & Prein, 2005; Lorenzen, 1996; Lorenzen & Enberg, 2002). Although this can be useful to prove a general relationship between the growth rate and an environmental factor, the a priori assumption of linearity is questionable.

Variation between cohorts arises due to differences in the growth history of cohorts. It is evident that cohorts in a given year differ in length due to the difference in age, but the length at a given age is likely to vary over time as well. This variation between cohorts might arise due to variation in the length at birth, but might also occur because cohorts lived at different times and therefore differ in the experienced environment (He & Bence, 2007; Wang & Thomas, 1995). A common way to correct for the growth history of individuals is to consider the average growth increment between two time points, instead of the actual length‐at‐age (Lipinski & Roeleveld, 1990; Rafail, 1973; Wang & Thomas, 1995). As we consider datasets that consist of independent length‐at‐age observations throughout years, this method can only be applied to the average length‐at‐age in every sampling instance and as such neglects individual variation in length‐at‐age and environmental limitation. In addition, this has been shown to yield less accurate estimates of the Von Bertalanffy growth parameters with a larger uncertainty (Vaughan & Kanciruk, 1982).

Similar to variation between cohorts, variation in length‐at‐age within cohorts arises due to differences in length at birth and differences in the experienced environment between individuals. Although individuals in the same cohort are not separated in time, they might be separated spatially or due to other ecological factors leading to variation in the experienced environment. In addition, genetic differences might cause variation between individuals as well. Consistent (genetic) differences between individuals can be accounted for by incorporating a random effect for every individual, but this is only feasible in datasets with multiple observations per individual. We focus on datasets with single observations for each individual, where this approach would only lead to extreme overfitting. Therefore, the only option to account for individual variation caused by a shared environment in datasets with single measurements is by considering the length‐at‐age at the population level as a distribution rather than a single value (Eveson et al., 2007; Pilling et al., 2002; Praineshu & Venugopalan, 1999).

The different sources of variation in the length‐at‐age are entangled due to the auto‐regressive nature of individual growth processes in which the current growth rate depends on the growth history of an individual. As a consequence, variation in growth arises between individuals and cohorts and could fluctuate over time. All these sources of variation should be considered to obtain an accurate estimate of the Von Bertalanffy growth parameters of a specific species, even if we are only interested in one of the sources or variation or average growth parameters. Such a method is currently unavailable. To fill this gap, we derive a model that describes the length distribution at a given age for every cohort, which can be used for datasets with a single length and age observation per individual. Because we derived this model from a stochastic version of the Von Bertalanffy growth equation for single individuals (Equation 1), it simultaneously includes variation due to changes over time, variation due to differences in the growth history of cohorts, and variation between individuals within a cohort. Here, we derive the model and apply it to length‐at‐age data of North Sea plaice (*Pleuronectes platessa*).

## 2 METHODS

### 2.1 Model formulation

We start with the equation describing the growth trajectory of a single individual (Equation 1). Individuals will differ in the experienced environmental limitation and this limitation might vary over time. We therefore assume that the limiting effect of the environment at a given point in time follows a Gaussian distribution of which the mean ($\mu_{t}$) and variance ($\sigma_{t}^{2}$) are allowed to vary over time:

(2) $$f_{t}∼N\left(\mu_{t},\sigma_{t}^{2}\right)$$

By substituting this distribution in Equation (1), we obtain a stochastic differential equation describing the growth of an individual born at time *T*_*b*_. We can solve this equation by separation of variables and integration:

(3) $$\begin{array}{l} d\ell_{\left(t,a,i\right)}=r_{B}\left(\mu_{t}\ell_{\infty }-\ell_{\left(t,a,i\right)}\right)\mathrm{dt}+r_{B}\sigma_{t}\ell_{\infty }{\mathrm{dW}}_{t}\\ \;\ell_{\left(T,a,i\right)}=\ell_{\left(T_{b},0,i\right)}e^{-r_{B}\left(T-T_{b}\right)}+\int_{T_{b}}^{T}r_{B}\mu_{t}\ell_{\infty }e^{-r_{B}\left(T-t\right)}\mathrm{dt}+\int_{T_{b}}^{T}r_{B}\sigma_{t}\ell_{\infty }e^{-r_{B}\left(T-t\right)}{\mathrm{dW}}_{t} \end{array}$$

The parameter $\ell_{\left(T_{b},0,i\right)}$ represents the length at birth of an individual. In addition, *W*_*t*_ represents a Wiener process, which describes the outcome of a continuous process with independent Gaussian increments ($W_{t+u}-W_{t}∼N\left(0,u\right)$). The integrals in this expression cannot be solved explicitly because the dynamics of the mean and variance of the environmental limitation ($\mu_{t},\sigma_{t}^{2}$) are not defined. If the environmental limitation was constant over time and space ($\mu_{t}=\mu ,\sigma_{t}^{2}=0$), individuals would follow a Von Bertalanffy growth curve toward a constant asymptotic length.

In this method, we consider datasets in which every individual is only measured once. In the ideal situation, these individuals are selected randomly from the population. In such datasets, it is not possible to follow the growth trajectory of a single individual and fit the derived growth curve on single individuals. Instead, we describe the distribution of the length‐at‐age for a cohort. Because we assumed that the environmental limitation of growth follows a Gaussian distribution, the length of individuals in a given cohort at time *T* follows a Gaussian distribution as well. Because the expected value of the Wiener process is equal to zero, we can derive an expression for the expected mean length at time *T* of a cohort born at time *T*_*b*_.

(4) $$\begin{array}{c} E\left[\ell_{\left(T,a\right)}\right]=E\left[\ell_{\left(T_{b},0,i\right)}e^{-r_{B}\left(T-T_{b}\right)}\right]+E\left[\int_{T_{b}}^{T}r_{B}\mu_{t}\ell_{\infty }e^{-r_{B}\left(T-t\right)}\mathrm{dt}\right]+E\left[\int_{T_{b}}^{T}r_{B}\ell_{\infty }\sigma_{t}e^{-r_{B}\left(T-t\right)}{\mathrm{dW}}_{t}\right]\\ \;=E\left[\ell_{\left(T_{b},a\right)}\right]e^{-r_{B}\left(T-T_{b}\right)}+\int_{T_{b}}^{T}r_{B}\mu_{t}\ell_{\infty }e^{-r_{B}\left(T-t\right)}\mathrm{dt} \end{array}$$

We omitted the indices referring to single individuals in the expression of the expected value of the length‐at‐age ($E\left[\ell_{\left(T,a\right)}\right]$), to make clear that this expected value is a statistic of the length‐at‐age distribution of a cohort rather than the length of single individuals. By using Equations (3) and (4) and applying Ito's isometry rule, we can also derive an expression for the expected variance in length at time *T* for a cohort born at time *T*_*b*_:

(5) $$\begin{array}{c} \begin{array}{c} V\left[\ell_{\left(T,a\right)}\right]=E\left[{\left(\ell_{\left(T,a,i\right)}-E\left[\ell_{T,a}\right]\right)}^{2}\right]=E\left[{\left(\int_{T_{b}}^{T}r_{B}\sigma_{t}\ell_{\infty }e^{-r_{B}\left(T-t\right)}{\mathrm{dW}}_{t}\right)}^{2}\right]\\ \;=E\left[\int_{T_{b}}^{T}r_{B}^{2}\sigma_{t}^{2}\ell_{\infty }^{2}e^{-2r_{B}\left(T-t\right)}\mathrm{dt}\right]=\int_{T_{b}}^{T}r_{B}^{2}\sigma_{t}^{2}\ell_{\infty }^{2}e^{-2r_{B}\left(T-t\right)}\mathrm{dt} \end{array} \end{array}$$

We assume that samples are taken with an approximately constant time interval and therefore discretize the equations characterizing the length distribution of a given cohort at time *T*. Under this assumption, the equations become independent of the length distribution at birth and can be applied without knowledge about the full growth history of a cohort.

(6a) $$\begin{array}{c} E\left[\ell_{\left(T+1,a+1\right)}\right]=E\left[\ell_{\left(T_{b},0\right)}\right]e^{-r_{B}\left(T-T_{b}+1\right)}+\int_{T_{b}}^{T+1}r_{B}\mu_{t}\ell_{\infty }e^{-r_{B}\left(T+1-t\right)}\mathrm{dt}\\ \;=E\left[\ell_{\left(T,a\right)}\right]e^{-r_{B}}+\int_{T}^{T+1}r_{B}\mu_{t}\ell_{\infty }e^{-r_{B}\left(T+1-t\right)}\mathrm{dt} \end{array}$$

(6b) $$\begin{array}{c} V\left[\ell_{\left(T+1,a+1\right)}\right]=\int_{T_{b}}^{T+1}r_{B}^{2}\sigma_{t}^{2}\ell_{\infty }^{2}e^{-2r_{B}\left(T+1-t\right)}\mathrm{dt}\\ \;=V\left[\ell_{\left(T,a\right)}\right]e^{-2r_{B}}+\int_{T}^{T+1}r_{B}^{2}\sigma_{t}^{2}\ell_{\infty }^{2}e^{-2r_{B}\left(T+1-t\right)}\mathrm{dt} \end{array}$$

To make these equations usable, we need to make assumptions about the dynamics of the mean and the variance of the environmental limitation ($\mu_{t}$ and $\sigma_{t}^{2}$). These quantities only appear within the integral from time *T* up to time *T* + 1. We therefore only have to make assumptions about the mean and variance of the environmental limitation between consecutive timepoints or measurements. We assume that the mean and variance of the environmental limitation between times *T* and *T* + 1 are well approximated by the average value of these quantities in the given time interval (${\bar{\mu }}_{T}$ and ${\bar{\sigma }}_{T}^{2}$). Under these assumptions, the model will approximate the growth dynamics if the interval between measurements becomes small relative to the average lifetime of an individual. Because we substitute the mean and variance of the environmental limitation by the average of these quantities over a time interval, they become independent of time in the domain of integration and we can solve the integrals in Equations (6a) and (6b), which results in the final form of our model:

(7a) $$E\left[\ell_{\left(T+1,a+1\right)}\right]=E\left[\ell_{\left(T,a\right)}\right]e^{-r_{B}}+{\bar{\mu }}_{T}\ell_{\infty }\left(1-e^{-r_{B}}\right)$$

(7b) $$V\left[\ell_{\left(T+1,a+1\right)}\right]=V\left[\ell_{\left(T,a\right)}\right]e^{-2r_{B}}+\frac{1}{2}r_{B}{\bar{\sigma }}_{T}^{2}\ell_{\infty }^{2}\left(1-e^{-2r_{B}}\right)$$

Interesting to note from this formulation is that the variance in environmental limitation over a given period (${\bar{\sigma }}_{T}^{2}$) has the same unit as the time constant (*T*). This arises because we model the length of an individual as a Brownian process which is a process with random increments. The variance of a Brownian process increases due to the random nature of the process and therefore depends on the length of the time between consecutive measurements in our model. In other words, the total variance in environmental limitation experienced by an individual increases (decreases) if the actual variation of the environment increases (decreases) or the individual experiences the environment for a longer (shorter) period of time.

To obtain a time‐independent measurement of the environmental variation, we can consider the long‐term asymptotic variation in individual length ($V\left[\ell_{\left(T,\infty \right)}\right]$). This represents the variation in length that individuals would have after spending an infinitely large time in an environment with a given amount of variation in growth limitation (${\bar{\sigma }}_{T}^{2}$). At this asymptotic variation in length, the loss of variation in length due to growth ($V\left[\ell_{\left(T,a\right)}\right]\left(1-e^{-2r_{B}}\right)$) is equal to the gain in variation in length due to variation in the environment ($\frac{1}{2}r_{B}{\bar{\sigma }}_{T}^{2}\ell_{\infty }^{2}\left(1-e^{-2r_{B}}\right)$). In other words, when a cohort reaches the asymptotic variation in length, the variation in length of a cohort does not change any further over time. From Equation (7b), we can therefore derive the expression of the long term asymptotic variance in length:

(8) $$V\left[\ell_{\left(T,\infty \right)}\right]=\frac{1}{2}r_{B}{\bar{\sigma }}_{T}^{2}\ell_{\infty }^{2}$$

### 2.2 Model application

The model proposed in Equations (7a) and (7b) predicts an independent Gaussian length distribution for every cohort at every discrete age and time value. Therefore, the model can be fitted to datasets containing pairs of age and length measurements using maximum likelihood estimates. The best results are obtained if individuals enter the population at approximately the same moment of the year and measurements represent a random sample of the population. This is especially important for individuals in the same age class and year. Assigning weights to the measurements allows to correct for biases in the sample, if biases are known. In addition, sample instances should approximately be evenly distributed in time and individual ages, and cohorts should be characterized on the same discrete scale as sample instances. For example, measurements could be taken yearly on randomly selected individuals at a specified date. The age of individuals is consequently measured in year classes and individuals born between two measurements belong to the same cohort. Fitting the model described by Equations (7a) and (7b) to a dataset with pairs of length and age measurements is done by optimizing the log likelihood through altering the value of the Von Bertalanffy scalar (*r*_*B*_), the length distribution at the youngest age at every time point ($E\left[\ell_{T,a_{\min}}\right]$, $V\left[\ell_{T,a_{\min}}\right]$), the length distribution of all other cohorts at the first time point ($E\left[\ell_{T_{\min},a}\right]$,$V\left[\ell_{T_{\min},a}\right]$) and the distribution of the environmental limitation between all time points (${\bar{\mu }}_{T}$, ${\bar{\sigma }}_{T}^{2}$), (Table 1). In the proposed model, the mean and variance of the environmental limitation always occur as a product with the maximum asymptotic length (${\bar{\mu }}_{T}\ell_{\infty }$, ${\bar{\sigma }}_{T}^{2}\ell_{\infty }^{2}$). Therefore, the maximum asymptotic length cannot be estimated separately with this method and is incorporated as a species‐specific scalar of the environmental limitation. We provided an R‐package (Croll, 2022) that includes a procedure for fitting the model to a dataset with pairs of age and length measurements using maximum likelihood optimization through optimization methods available in the NLoptR‐package (Johnson, 2022).

**TABLE 1:** Description of the parameters that are estimated during the model fitting procedure.

Parameter	Description	Type	Number of parameters
*n*_*T*_	Number of sampling instances	Discrete	0 (fixed value)
*n*_*a*_	Number of sampled age classes	Discrete	0 (fixed value)
*T*_min_	Time of the first sampling instance	Discrete	0 (fixed value)
*a*_min_	First age class in the dataset	Discrete	0 (fixed value)
*r*_*B*_	Von Bertalanffy growth rate scalar	Continuous	1
$E\left[\ell_{\left(T,a_{\min}\right)}\right]$	Expected mean length at the first age class	Continuous	*n*_*T*_
$V\left[\ell_{\left(T,a_{\min}\right)}\right]$	Variance in length at the first age class	Continuous	*n*_*T*_
$E\left[\ell_{\left(T_{\min},a\right)}\right]$	Expected mean length at the first sampling instance	Continuous	*n*_*a*_−1
$V\left[\ell_{\left(T_{\min},a\right)}\right]$	Expected variance in length at the first sampling instance	Continuous	*n*_*a*_−1
${\bar{\mu }}_{T}\ell_{\infty }$	Mean asymptotic length	Continuous	*n*_*T*_−1
${\bar{\sigma }}_{T}^{2}\ell_{\infty }$	Variance in asymptotic length	Continuous	*n*_*T*_−1

The R‐package for fitting the described Von Bertalanffy growth model contains some additional features to tailor the model to specific populations. The first feature deals with the dynamics of the mean length at age when the environmental limitation is very variable. In the model described in Equations (7a) and (7b), the mean length of a cohort ($E\left[\ell_{T,a}\right]$) decreases if it exceeds the asymptotic length at some time step (${\bar{\mu }}_{T}\ell_{\infty }$). This is a mathematical artifact of the model that predicts that individuals will shrink in size if they are too large to be supported by the environment. Although some species might shrink in size under bad conditions, this is not realistic for all species (Kooijman, 2010). This could be solved by either assuming a log‐normally distributed error structure or by simply fixing the change in the mean length at age to non‐negative values. The first option is mathematically very complex. Instead, the package includes a version of the model in which the average length of a cohort does not decrease if the average cohort length exceeds the maximum asymptotic length. This version of the model should be used with care and only with reasonable arguments, because this method inflates the impact of small and younger cohorts on the estimated environmental limitation. In any case, we advise to first fit the model without this additional assumption, to check whether this indeed predicts large decreases in mean length of some cohorts.

The second extension available in the R‐package deals with differences in environmental limitation between age and length classes. Differences in environmental limitation between age or length classes can arise if age or length classes show spatial segregation or differ in diet. The model allows specification of age or length classes and estimates separate means and variances in environmental limitation (${\bar{\mu }}_{T,c}$, ${\bar{\sigma }}_{T,c}^{2}$) for every age or length class at every time step. It is important to note that this extension of the model only uses the mean length of a cohort to identify the length class and therefore all individuals in a cohort are always placed in the same length class. In addition, the number of observations per class decreases with an increase in the number of age or length classes. The incorporation of age or length classes can therefore make the model fit less accurate if there are no true differences in the environmental limitation of the selected classes.

### 2.3 Application to North Sea plaice

To illustrate the use of the proposed model, we fit the model to a dataset with age and length measurements of plaice (*Pleuronectes platessa*) obtained from the Beam Trawl survey (BTS). This survey is designed to monitor plaice in the North Sea and is consistently conducted in the third quarter (July to September) from 1990 onwards. The length of individuals is measured with at least 5 mm accuracy and the age of sampled individuals is obtained through otolith readings. We downloaded the datasets with individual ages and lengths recorded during the third quarter of 1990 to 2021 from the online ICES DATRAS data portal on the 1st of November 2021 (ICES, 2021). The size distributions in the dataset are likely to be skewed due to size‐dependent mortality in the population and biases in the sampling process. Differences between age classes and years are unlikely to affect the estimated size distributions because the model estimates a size distribution separately for every age in every year. In contrast, differences between the size classes are likely to skew the size distribution of a given age. The age–length observations were therefore weighted by the inverse of the catch per unit effort (CPUE) of the observed length. The CPUE per length indicates the probability that an individual of a given length is caught in the survey of a given year. Weighting the observations with the inverse of the CPUE corrects for any factor that affects the catch probability of a given length in a given year. After weighing of the samples, all lengths approximately had the same contribution to the dataset, and visual inspection confirmed that the data approximated the assumption that length at age in a given year follows a Gaussian distribution (judged by eye, Figure A3 in Appendix A).

Starting values for the expected length at the first age in the dataset and the expected asymptotic length and the growth scalar were estimated by fitting a Von Bertalanffy growth model without considering differences between years and cohorts ($E\left[\ell_{\left(T,a_{\min}\right)}\right]=120\;\mathrm{mm}$, ${\bar{\mu }}_{t}\ell_{\infty }=380\;\mathrm{mm}$ and $r_{B}=0.303y^{-1}$). Starting values for the variance in length at the lowest age class and the variance in asymptotic length were set to the variance in the youngest and oldest age class, respectively ($V\left[\ell_{\left(T,a_{\min}\right)}\right]=801\;{\mathrm{mm}}^{2}y$ and ${\bar{\sigma }}_{t}^{2}\ell_{\infty }^{2}=5789\;{\mathrm{mm}}^{2}y$).

We used the Sbplx algorithm of the NLoptR‐package (Johnson, 2022), which is a variant of the Nelder–Mead optimization method, with a relative tolerance of 10^−10^ to optimize the likelihood of our model. The optimization was performed using a log‐transformed parameter space to account for the magnitudinal difference between parameters. For comparison, we fitted two versions of the model. In the first version, the environmental limitation was constant over years and therefore the mean and variance of the asymptotic length were estimated as a single parameter. In the second version, the environmental limitation was allowed to vary between years, and the mean and variance of the asymptotic length were estimated separately for every year.

To assess the robustness of the model with yearly varying asymptotic length, we used a jackknife approach in which we repeated the analysis 31 times with data from one entire year omitted every time. This shows the impact of the samples from a given year on the model fit and gives an indication of the robustness of the method to years in which no data could be collected. Lastly, we demonstrate the use of separate age groups with different environmental limitation in the model by splitting the plaice population in three ecological groups by age.

We used a estimation of the maximum asymptotic length ($\ell_{\infty }=780$) estimated by Van der Veer et al. (2001) and scaled the estimated mean and variance in asymptotic length with this value to obtain the mean and variance in environmental limitation (${\bar{\mu }}_{t}$,${\bar{\sigma }}_{t}^{2}$).

## 3 RESULTS

**FIGURE 1:**
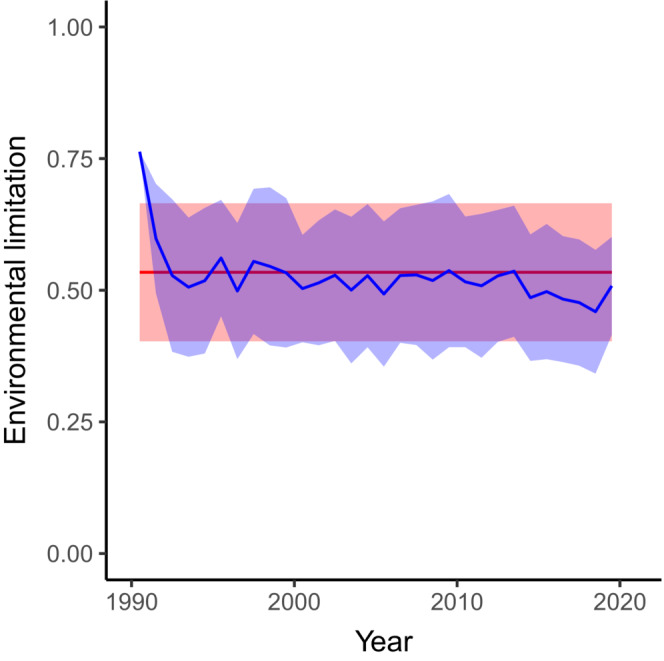
Fitted environmental limitation (${\bar{\mu }}_{T}$) for the model with constant environmental limitation (red) and the model with yearly varying environmental limitation (blue). Shaded areas indicate the mean plus or minus the two times standard deviation derived from the estimated asymptotic variances ($\frac{1}{2}r_{B}{\bar{\sigma }}_{T}^{2}$).

A model with a constant environmental limitation and a model with yearly varying environmental limitation were fitted to a length‐at‐age dataset for North Sea plaice. The model with yearly varying mean and variance of the environmental limitation fitted the data better compared to the model with constant mean and variance of the environmental limitation (AIC of respectively 16,076,750 and 16,048,031, likelihood ratio test: *p* < .001). This suggests that the mean and variance of the environmental limitation are likely to fluctuate between years. More precisely, the model with yearly varying environmental limitation suggests a weak downward trend in this limitation, indicating that the environmental limitation became stronger over time (Figure 1, solid line). The estimated variance in the environmental limitation is slightly larger if the environmental limitation is fixed compared to the model in which the environmental limitation is allowed to fluctuate (0.0335*y* and on average 0.0268*y*, respectively). This overestimation of the variance in environmental limitation arises because the fixed limitation model accounts for the variation in the asymptotic length between years in addition to the variation in asymptotic length within a year. As expected, the models also differ slightly in the estimated parameters defining the length distribution at the youngest age. The estimates of the Von Bertalanffy growth scalar of the models (0.2553*y* ^−1^ and 0.2977*y* ^−1^, respectively) are relatively close to estimates based on individual energy expenses (0.2955*y* ^−1^, Van der Veer et al., 2001).

**FIGURE 2:**
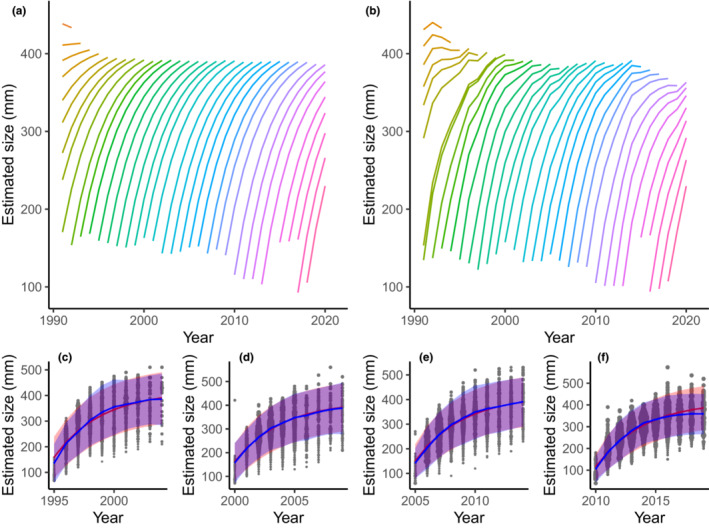
Expected value of the length‐at‐age ($E\left[\ell_{\left(T,a\right)}\right]$) for the model with a constant environmental limitation (a) and a yearly varying environmental limitation (b). Line colors correspond to the year of birth of the cohort. The expected length‐at‐age for the cohorts born in the years 1995 (c), 2000 (d), 2005 (e), and 2010 (f) are plotted separately for the model with a constant environmental limitation (red) and the model with a yearly varying environmental limitation (blue), together with the data points corresponding to the specific cohort. Shaded areas indicate the expected length plus or minus two times the standard deviation from the estimated length distribution. The size of the data points indicates the weighted number of observations of a specific age–length combination in the given cohort ranging from 1 to 60 times.

The estimated model parameters lead to predictions of the length‐at‐age distribution for every cohort, which differ most strongly for the older age classes (Figure 2). On visual inspection, both the model with a constant environmental limitation and a yearly varying environmental limitation appear to fit the datapoints well (Figure 2c–f). Note that the expected length of individuals in a cohort can shrink in the model with yearly varying environmental limitation. This occurs if the estimated mean asymptotic length falls below the expected length of individuals in a cohort (${\bar{\mu }}_{T}\ell_{\infty }<E\left[\ell_{\left(T,a\right)}\right]$). While such a decrease can realistically occur, it is sometimes a biologically impossible result. Repeating the analysis on this dataset with the restriction that the expected length of a cohort cannot decrease, yields very similar results (not shown). Nonetheless, this additional restriction should be handled with care, because early tests on simulated data showed that this restriction makes the model more dependent on the data points of young age classes.

**FIGURE 3:**
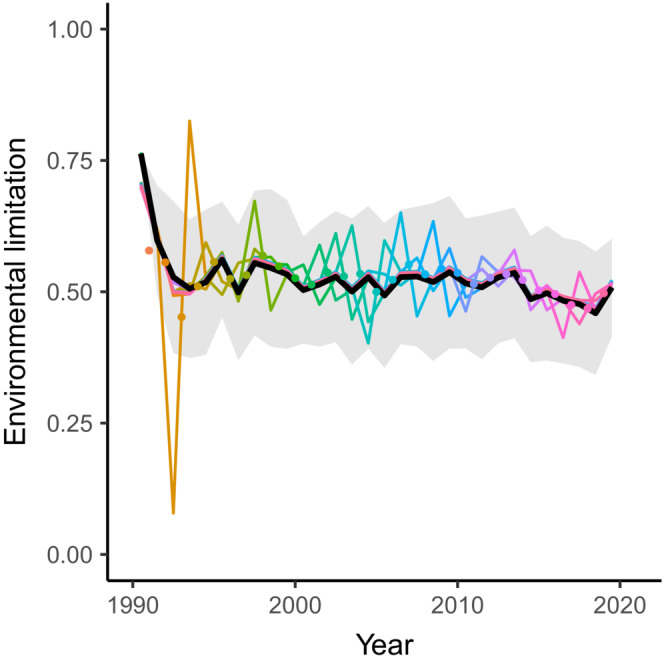
Fitted environmental limitation (${\bar{\mu }}_{T}$) as predicted during a jackknife approach. Colors correspond to different model fits. For every model fit, the data in the year indicated by the dot is omitted from the analysis. The black line is the estimated mean environmental limitation without omitted data and shaded areas indicate the mean plus or minus two times standard deviation derived from the estimated asymptotic variances without omitted data ($\frac{1}{2}r_{B}{\bar{\sigma }}_{T}^{2}$).

To demonstrate the robustness of the model, we used a jackknife approach in which we repeated the analysis with the samples from 1 year omitted (Figure 3). It is not unlikely that actual datasets will contain years for which there is no data, for example, due to sampling problems. This analysis showed that missing samples mainly affect the estimate of the mean environmental limitation in the time step directly before and directly after the sample instance with missing data. At one of these time steps, the mean environmental limitation will be overestimated while it will be underestimated in the other time step. In addition, it seems that this over‐ and underestimation of the mean asymptotic length becomes larger toward the start and end of the time period included in the model. A possible cause for this pattern is that these time steps include cohorts which partly fall outside the time period covered by the data and therefore are estimated on a restricted number of ages. This could make estimates of the growth curves of these cohorts more vulnerable to missing data, which is reflected in the larger over‐ and underestimations of the asymptotic length in the years these cohorts are in. Indeed, the early years included in the analysis include significantly less observations than later years. Lastly, it is clear that the over‐ and underestimation of the mean asymptotic length due to omitted data is small compared to the variation between individuals within a cohort.

**FIGURE 4:**
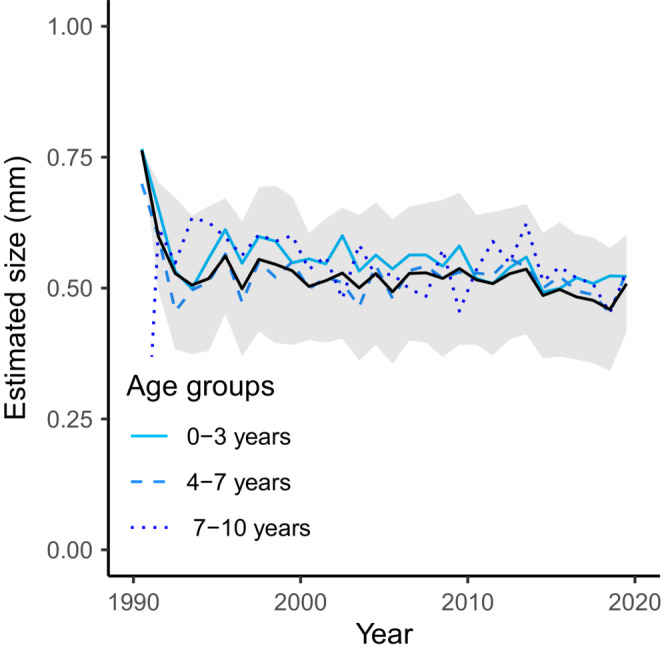
Estimated environmental limitation if three groups with separate environmental limitations are defined based on age. The black line and shaded area indicate the mean plus or minus the two times standard deviation derived from the estimated asymptotic variances if all ages are part of the same group ($\frac{1}{2}r_{B}{\bar{\sigma }}_{T}^{2}$).

Our estimation method can also be used on populations which consist of separate ecological groups. We demonstrate this using plaice, the distribution of which has been shown to shift away from the coast with increasing length or age (Basimi & Grove, 1985; Braber & De Groot, 1973; Rijnsdorp & Vingerhoed, 2001). We divide the plaice population into three arbitrary age groups to represent this spatial shift with age, respectively a group up to 3 years old, a group from 4 to 7 years old, and a group with individuals over 7 years old. The model fit yields an estimate of the mean and the variance of the environmental limitation for every year and group (Figure 4). The model with three age groups does indeed fit the data better compared to the model without age groups (AIC of respectively 15,982,917 and 16,048,036). Despite a similar trend, year to year changes in some years differ substantially between length groups, both in magnitude and direction. Such differences could indicate relevant ecological differences between the groups. The average estimated variance in environmental limitation for the youngest age group (on average 0.0217*y*) is smaller compared to the average estimated variance in environmental limitation in the model without age groups (on average 0.0268*y*). This might suggest that the environmental limitation of individuals in the youngest age group is more similar to the environmental limitation of individuals in the same age group compared to the environmental limitation of individuals in other age groups. This could explain why the model with three age groups fits the data better compared to the model without age groups. In contrast, the average estimated variance in environmental limitation for the two oldest age groups (on average 0.0399*y* for age group 4–7 years and 0.0801*y* for age group 7–10 years) is larger compared to the average estimated variance in environmental limitation in the model without age groups (on average 0.0268*y*). This might suggest that the environmental limitation of some individuals in the two oldest age groups is more similar to the environmental limitation of individuals in other age groups compared to the environmental limitation of individuals in the same age group. It is important to note that the reported variation corresponds to the fitted variation in environmental limitation and not the variation in the individual sizes. The variation in individual size is a balance between the variation in size at the previous time step and the variation in environmental limitation. The variation in size is therefore likely to increase or decrease with age, depending on the variation in size at birth. This does not hold for the variation in environmental limitation as it is independent of size and age.

## 4 DISCUSSION

We presented a new method to estimate Von Bertalanffy growth parameters from datasets with pairs of age and length measurements and provide an R package called VBGfit (Croll, 2022) to apply this method. The method is based on a model that describes the length distribution of cohorts in a population under the assumption that cohorts partly overlap in time and experience a fluctuating environment (Equations 7a and 7b). The model is derived from a stochastic differential equation describing the growth of a single individual in a fluctuating environment (Equation 1) and therefore accounts for variation due to changes in the environment over time, variation in the growth history of cohorts and variation between individuals within a cohort. Because the model is described in a discretized form, it is easy to fit on pairs of length and age measurements taken with a regular interval, which is one of the most common forms of data on population structure (Eveson et al., 2007).

Our model makes several assumptions about the underlying population structure to obtain a model applicable to datasets with random observation pairs of individual lengths and ages. First of all, we assume individuals follow a Von Bertalanffy growth curve in which only the asymptotic length fluctuates over time and between individuals (Equation 1). This is the most common and first proposed form of the Von Bertalanffy growth equation (von Bertalanffy, 1938). Nonetheless, it is sometimes assumed that both the asymptotic length and the Von Bertalanffy growth rate scalar fluctuate (Eveson et al., 2007; Pilling et al., 2002). It has been shown that estimates of the asymptotic length and the Von Bertalanffy growth rate scalar are strongly correlated if both are allowed to fluctuate. Due to this correlation, it might be difficult to obtain correct parameter estimates, because different sets of parameters are likely to fit the dataset equally well (Eveson et al., 2007; Pilling et al., 2002). In addition, it has been shown that only the asymptotic length or the Von Bertalanffy growth rate scalar has to fluctuate to obtain a very good prediction of the population structure and an accurate estimate of the environmental limitations, when data consists of independently observed pairs of individual age and length (Eveson et al., 2007). We therefore chose to only make the asymptotic length dependent on the environmental limitation, as this has the most comprehensive substantiation in energetic theory (Kooijman, 2010). Secondly, we assume that the dynamics of the environmental limitation between two measurements can be described accurately by the average environmental limitation in this period. This is a very convenient assumption borne from the discrete nature of most datasets with length‐at‐age data. Nonetheless, it is possible to substitute a more complex, time‐dependent formulation for the environmental limitation in Equations (6a) and (6b) and work out the more complex model through integration. This would lead to a more specific and less generally applicable form of the model. Thirdly, we assume that the environmental limitation experienced by an individual at a given moment is drawn from a Gaussian distribution. The central limit theorem states that if a variable is influenced by many additive random factors, it will approach a Gaussian distribution. As the environmental limitation emerges from a complex ecological, chemical, or physical system, it is likely to be influenced by many random factors and therefore is likely to approach a Gaussian distribution. In conclusion, most of the assumptions in this method are made to ensure that the model is as generic as possible but still applicable to the currently available datasets with length‐at‐age data.

**FIGURE A1:**
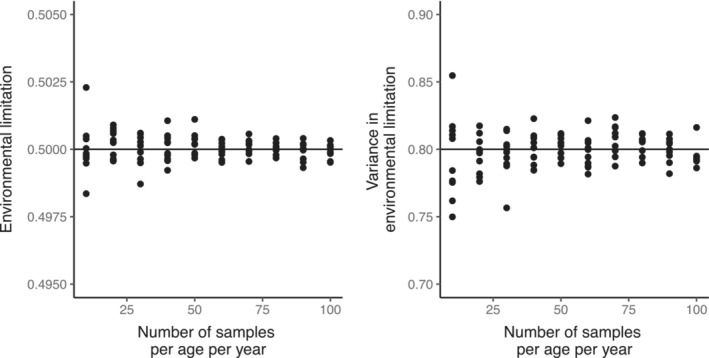
Average of the estimated mean and variance of the environmental limitation for different number of data points drawn from a simulated population structure. The black line indicates the actual values of the mean and variance in environmental limitation in the simulation.

**FIGURE A2:**
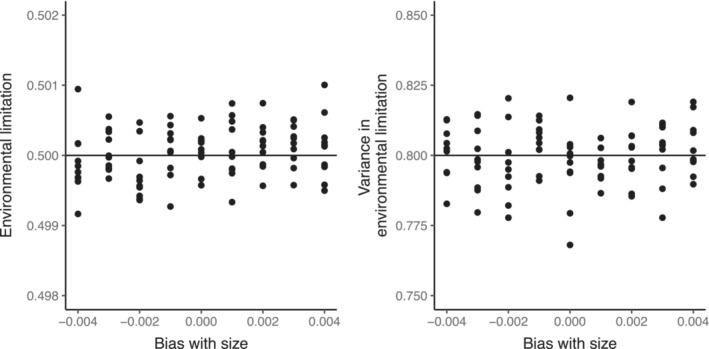
Average of the estimated mean and variance of the environmental limitation when the data contains a bias with length. A positive bias with length indicates that larger individuals contribute more to the samples, while a negative bias with length indicates that smaller individuals contribute more to the samples. The black line indicates the actual values of the mean and variance in environmental limitation in the simulation.

**FIGURE A3:**
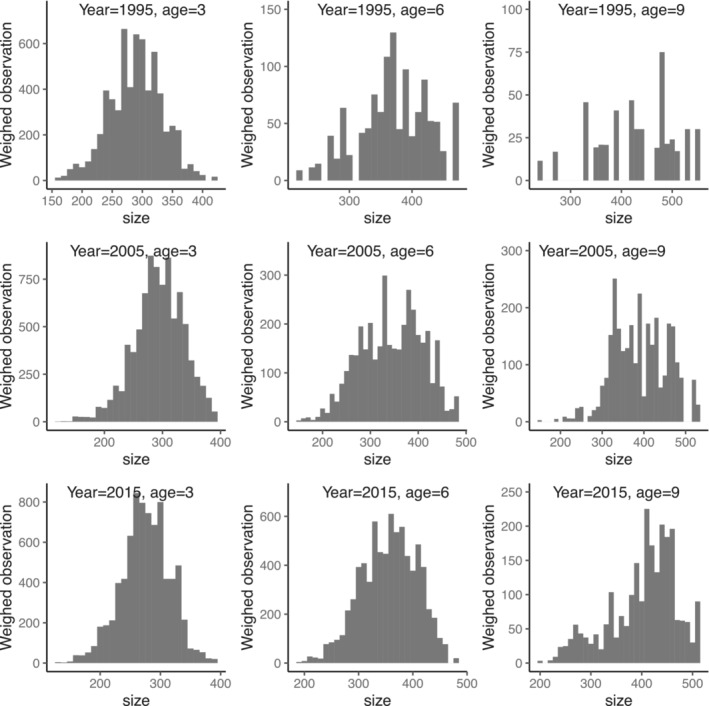
Weighted distributions of length at age at a given year. The number of observed individuals with a specific length is weighted by the inverse of the catch per unit effort of that length in the given year. The length at age seems to approximately follow a normal distribution.

Just like other methods, our model assumes that individual length is normally distributed in a cohort and therefore in the obtained samples. Deviations from this normal distribution can occur for example due to sampling biases or a link between individual mortality rate and individual length. Fits on simulated data showed that the value of mean environmental limitation is slightly overestimated if the contribution to the data increases with size, while the value of the mean environmental limitation is underestimated if the contribution to the data decreases with size (Figures A1 and A2 in Appendix A). This is comparable to a situation in which larger or smaller individuals respectively have a higher probability of ending up in the data regardless of their age. These model fits on simulated data show that the effects of length bias in the data might be relatively small. Nonetheless, it is important to correct for skewness in the individual length distribution in the data when possible. One way to do so is to add relative weights to the samples. In our example with North sea plaice, we weighted the samples by the inverse of the catch per unit effort (CPUE) per length. The CPUE is a measure of the relative presence of a length class in the dataset in a given year. In this way, we corrected for the impact of length on the catchability of an individual, which can arise for example due to very strong length‐specific mortality or harvesting probabilities. This resulted in a dataset in which individual length approximates a normal distribution of every age class in every year (Figure A3 in Appendix A). Because our model allows to add weights to every individual sample, it is in theory possible to correct for biases linked to any trait of an individual including age and size. Nonetheless, it is important to note that it is not possible to correct for all biases because specific information is often lacking. Especially biases in growth due to genetic differences and habitat quality might require attention as these could directly impact growth and skew the size distributions in a population.

A novel and very important aspect of our method is that it accounts for variation caused by environmental changes over time, variation between the growth history of cohorts and variation between individuals within a cohort simultaneously. Earlier methods only account for variation due to changes over time by fitting a growth curve separately for every sampling instance, or only account for the growth history of a cohort by fitting a growth curve separately for every cohort. Similarly, more recent methods only accounted for variation between individuals (He & Bence, 2007; Pilling et al., 2002; Praineshu & Venugopalan, 1999; Rafail, 1973; Vincenzi et al., 2014; Wang & Thomas, 1995) or variation caused by changes through time (Cloern & Nichols, 1978; Lorenzen, 1996; Lorenzen & Enberg, 2002). Due to the autoregressive nature of individual growth rates, these sources of variation are strongly intertwined and should not be considered separately. We account for this by fitting the Von Bertalanffy growth equation for all cohorts and sampling instances simultaneously. Because we derived our model from a stochastic differential equation describing individual growth, our model also accounts for variation in environmental limitation between individuals. In addition, we show that the variation between individuals is overestimated if a model does not account for changes in environmental limitation over time.

Because our model simultaneously accounts for variation between individuals and cohorts and allows variation from the environment to fluctuate over time, the model can be used for a wide range of applications. First of all, the estimated mean and variance of the asymptotic length estimated by our model can be used as a summary statistic for environmental limitation under the assumption that the sources of variation are independent. Growth of individuals is likely limited by numerous factors, which are often unknown. Our method offers a summary statistic for the cumulative distribution of all these factors. Our method is especially appropriate to estimate individual limitation in growth due to limitation through food availability. General theory about individual energy allocation links the asymptotic length of this Von Bertalanffy growth equation to the energy ingestion by individuals (Kooijman, 2010; von Bertalanffy, 1938). The distribution of the environmental limitation estimated by our model could therefore be used as a proxy for the distribution of food availability among the individuals in a population. Estimates of individual food availability are scarce, because they commonly have to be obtained from intensive observations or analysis of stomach samples. Because our model provides a proxy of the individual food availability throughout the entire population, it can be used for more detailed analyses of the dynamics of food availability. For example, linking the estimated environmental limitation to the consumer density might reveal density dependent feedbacks in the growth rate of individuals. The environmental limitation as a proxy for individual food availability might also provide insight into feeding links between species. The environmental limitation in our model always appears as a product with the maximum asymptotic length ($f_{t}\ell_{\infty }$); it should therefore first be scaled by an estimate of the species specific maximum asymptotic length before it can be compared between species. Comparison of this scaled proxy for individual food availability between species might then reveal links such as shared resources or competition.

In our example with North sea place, we showed that our model can be used to explore environmental segregation between length or age groups as well. The R‐package (Croll, 2022) allows to split a population into a priori defined length or age groups and fits an environmental limitation separately for every length or age group. With a realistic division in length or age groups, our model could provide valuable information about growth limitation in different life stages. If for example length or age groups show very diverse patterns in environmental limitation, it is likely that the length or age groups are environmentally separated either through segregation in space or differences in diet. In this way, the model could therefore yield additional understanding in the growth dynamics during various life stages.

It is often difficult to judge whether a certain division in length or age groups is valid. A way to assess the suitability of a division in age or length groups is to look at the estimated variance in environmental limitation for every group compared to the variance in environmental limitation estimated in a fit without groups. Without groups, our general model combines all length and age groups and fits a single environmental limitation for all groups. This method lumps together the variation in environmental limitation within and between age or length groups which will lead to a high estimate of the variance in environmental limitation if groups strongly differ in environmental limitation. In general, one can assume that individuals within a group are more similar to each other compared to individuals within another group. The variance in environmental limitation estimated for a single group is therefore expected to be lower compared to the variance in environmental limitation estimated for the entire population. This was only the case for the youngest age group (up to 3 years) in our example of North Sea plaice. This suggests that our arbitrary division of the population into three age groups does not accurately represents the ecology of North Sea plaice. It does demonstrate that our model can be used to verify whether a suspected division in ecological groups is likely by comparing the estimated variance in environmental limitation of a fit without ecological groups with a fit with ecological groups as is done in the example for north sea plaice.

Lastly, our method might also be applicable to management as it is able to model variation in growth through time and between individuals based on only a limited number of parameters (Flinn & Midway, 2021). Many management models, in particular those used to estimate reference points for fish stock management, assume a fixed length distribution at a given size, while variation in growth rates is shown to be important for the response of populations to exploitation (Lorenzen & Enberg, 2002). With our model, the variation in growth between years can be easily quantified, resulting in a more accurate prediction of the length at age for every cohort. The age–length relationships from our model then can be used to calculate a more precise estimate of the needed reference points.

In conclusion, our model provides a way to estimate growth curves and length distributions of individual cohorts based on single individual length and age observations. In our model, growth is allowed to vary over time, while our model also accounts for variation between individuals and variation between cohorts. So far, these factors could only be estimated simultaneously if the growth history of individuals was known. Models for single observations of individual length and age only accounted of a subset of these factors. Our model does account for all these factors and in this way estimates a proxy for the limitation in individual growth, which may vary over time. This estimate of the limitation in individual growth is a new step in understanding patterns in individual growth based on individual field observations.

## AUTHOR CONTRIBUTIONS

**Jasper Cornelis Croll:** Conceptualization (equal); formal analysis (lead); methodology (lead); software (lead); writing – original draft (lead); writing – review and editing (equal). **Tobias van Kooten:** Conceptualization (equal); funding acquisition (lead); supervision (lead); writing – review and editing (equal).

## CONFLICT OF INTEREST

The authors declare that there is no conflict of interest.

## Data Availability

The R package to perform the analysis is available through the following https://doi.org/10.5281/zenodo.6797840. The data used for the North Sea plaice example is publicly available on ICES DATRAS data portal: https://datras.ices.dk.

## APPENDIX A

### Model Test on Simulated Data

We fitted the model to simulated data to test the sensibility of the estimated parameters to the number of observations in the data and possible biases in length in the data. To do so, we generated the population structure of a population with a constant mean and variance of the environmental limitation. To do so, we used the following continuous model:

(A1)
$$\frac{\partial E\left(\ell \right)}{\partial t}+\frac{\partial E\left(\ell \right)}{\partial a}=r_{B}\left(\mu_{f}\ell_{\infty }-E\left(\ell \right)\right)$$

(A2)
$$\frac{\partial V\left(\ell \right)}{\partial t}+\frac{\partial V\left(\ell \right)}{\partial a}=2r_{B}\left(\sigma_{f}^{2}\ell_{\infty }^{2}-V\left(\ell \right)\right)$$

The average length at birth was determined by drawing a random value from a predetermined interval ($\ell_{0\min}$–$\ell_{0\max}$). Other parameters of the simulation were fixed (Table A1). The population structure was simulated over a period of 30 years and every cohort was simulated from age 0 to 10. A sample was drawn from the population structure for every year and cohort at 180 days after a new cohort with age 0 is introduced to the population.

**TABLE A1:** Parameters used for simulating population structure.

Parameter definition	Symbol	Value	Unit
Von Bertalanffy growth rate scalar	*r*_*B*_	0.001	*d* ^−1^
Maximum asymptotic length	$\ell_{\infty }$	1000	mm
Mean environmental limitation	$\mu_{f}$	0.5	‐
Variance in environmental limitation	$\sigma_{f}^{2}$	0.0002	‐
Minimum length at birth	$\ell_{0\min}$	5	mm
Maximum length at birth	$\ell_{0\max}$	15	mm
Maximum cohort age	‐	3650	*d*
Time between cohort starts	‐	365	*d*
Time between cohort start and sampling	‐	180	*d*

To test the influence of the number of parameters on the estimated environmental limitation, we draw 100 datasets from the simulated population structure. The number of samples varied between 10 and 100 samples per cohort per year (Figure A1). The estimated mean and variance of the environmental limitation are distributed evenly around the actual mean and variance in the simulation. With an increasing number of samples, the standard error of the estimated mean and variance of the environmental limitation become smaller. In other words, the estimated mean and variance in environmental limitation become on average closer to the real mean and variance if the number of samples increases.

Another concern when estimating environmental limitations from sampled length structures is a possible bias in the sample method. It is possible that the samples are biased toward individuals with a larger of smaller length. To test the effect of this bias on the estimated environmental limitation, we sampled 100 data sets with 50 observations per cohort per year from the simulated population structure. When fitting our model, we weighed the samples by individual length. The weight of a sample changed linearly with individual length ($w_{i}=a\ell_{i}+b$). The slope of this relationship determined the strength of the bias, ranging from a sampling bias towards larger individuals (*a* > 0) to a sampling bias towards smaller individuals (*a* < 0). The intercept of the relation between bias weight and length is determined in such a way that the average weight of samples is equal to one ($b=1-\frac{\sum_{i=1}^{n}a\ell_{i}}{n}$). This will ease comparison between model fits, because the weighted number of samples is equal for all datasets.

A sample bias linked to weight might result in an error in the estimation of the mean environmental limitation, but does not affect the estimation of the variance in environmental limitation (Figure A2). The mean environmental limitation is slightly overestimated if larger individuals are more likely to end up in the samples, while the mean environmental limitation is slightly underestimated when smaller individuals are more likely to end up in the sample. Although this error seems to be small, it is important to keep this bias in mind an correct for this bias when possible.
